# Muscle‐Derived Bioactive Factors: MyoEVs and Myokines

**DOI:** 10.1111/cpr.13801

**Published:** 2024-12-30

**Authors:** Xupeng Liu, Ziyue Yao, Liping Zhang, Ng Shyh‐Chang

**Affiliations:** ^1^ Key Laboratory of Organ Regeneration and Reconstruction, State Key Laboratory of Stem Cell and Reproductive Biology Institute of Zoology, Chinese Academy of Sciences Beijing China; ^2^ Institute for Stem Cell and Regeneration, Chinese Academy of Sciences Beijing China; ^3^ University of Chinese Academy of Sciences Beijing China; ^4^ Beijing Institute for Stem Cell and Regenerative Medicine Beijing China

## Abstract

Overview of the functions and applications of myokines and MyoEVs.

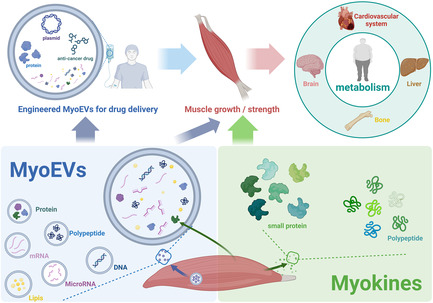

To the Editor,

Skeletal muscles are not only responsible for movement and energy metabolism, but are also increasingly recognised as an important endocrine organ [1]. Skeletal muscles secrete various bioactive substances, including muscle‐derived extracellular vesicles (MyoEVs) and myokines. This letter explores the functions, mechanisms of action and applications of MyoEVs and myokines. By comparing their composition, secretion pathways, transport mechanisms, range of action, functional differences and clinical applicability, we aim to highlight their crucial roles in regulating skeletal muscle function and physiological homeostasis, as well as their respective advantages and limitations in disease treatment.

## MyoEVs: Definition, Classification and Mechanisms of Action

MyoEVs are small vesicles secreted by skeletal muscle cells, with a bilayer phospholipid and protein shell, containing a variety of biomolecules, including proteins, lipids and complex RNAs [2, 3]. Based on differences in their biogenesis, release mechanisms and functions, they can be classified into exosomes, microvesicles and apoptotic bodies [3]. Exosomes are small EVs (30–150 nm in diameter) that are formed through the inward budding of the endosomal membrane and are released upon the fusion of multivesicular bodies with the plasma membrane [2, 4]. During muscle contraction or exercise, muscle cells secrete large amounts of exosomes [5, 6, 7, 8], to exchange bioactive substances and signals with both adjacent and distant target cells. The diverse cargo and mechanisms of action of MyoEVs make them important mediators of muscle‐to‐muscle and muscle‐to‐other‐tissue communication, contributing to processes such as muscle development, regeneration and adaptation to exercise [9, 10].

## Myokines: Definition, Types and Mechanisms of Action

Myokines are cytokines or other proteins secreted by skeletal muscle, typically small proteins or peptides, in response to stimuli such as physical activity or exercise [11, 12]. Some studies also include RNAs, but in here, myokines are considered to include only small proteins and peptides. Common myokines include IL‐6, FGF21, Irisin, Follistatin, IGF1, BDNF and VEGF [11, 12].

Like MyoEVs, the mechanisms by which myokines exert their effects on target tissues involve both local and systemic pathways. Locally, myokines can act in an autocrine or paracrine manner, directly influencing the function and metabolism of skeletal muscle cells. Systemically, myokines can be released into the bloodstream and act on distant target tissues, such as the liver, adipose tissue and the brain, to regulate various physiological processes. Myokines play a crucial role in the progression of various diseases [13, 14]. For instance, in muscle atrophy (such as Duchenne muscular dystrophy), the abnormal expression of certain Myokines like IL‐6 may mediate muscle damage. In metabolic diseases such as diabetes and cardiovascular diseases, myokines may be potential therapeutic targets by regulating the systemic metabolic environment.

## Comparison of MyoEVs and Myokines

### Differences in Secretion, Transport Mechanisms and Range of Action

Myokines, as constituents of the secretome of skeletal muscle, are predominantly secretory proteins, which typically exert their effects through autocrine, paracrine, or endocrine mechanisms [11, 12]. Most myokines identified to date are soluble proteins or peptides that initiate downstream signalling by binding to specific receptors, thereby regulating the gene expression and metabolic activities of target cells [1, 11, 12]. The cellular secretion pathway directed by N‐terminal signal peptides is a crucial factor for myokines to enter the circulation and fulfil their biological functions through cellular communication and inter‐tissue communication. For instance, irisin, which responds to exercise, is derived from the transmembrane protein FNDC5 through proteolytic cleavage, releasing the extracellular domain to form irisin, which is then released into the bloodstream as a secreted protein to exert its effects.

Unlike Myokines, MyoEVs are membrane‐enclosed vesicles capable of protecting and delivering a variety of signalling biomolecules with different solubilities (including microRNAs, tRNAs, mRNAs, proteins and lipids) and directly exchanging materials with local or distant cells [2, 3, 9, 15]. At the same time, MyoEVs can regulate gene expression and cellular functions through various mechanisms such as receptor binding, endocytosis, direct fusion. And, there is a distinction in the protein species covered by myokines and MyoEVs. The proteins loaded within MyoEVs include not only secretory proteins but also a substantial amount of functional intracellular proteins. Previous proteomic data analysis of MyoEVs revealed that secretory proteins constitute only about 35% of the total protein types in MyoEVs. Moreover, the proteins in MyoEVs do not seem to include the classic known myokines, such as Myostatin, LIF, Apelin, IL‐4, IL‐6, IL‐7, IL‐15, irisin, Musclin and so on [16]. Lipids are an important part of MyoEVs, and molecules such as sphingomyelin, hexose ceramide, phosphatidylinositol and free cholesterol found in MyoEVs can be used as biomarkers of muscle diseases [15]. However, most extant studies have focused on the role of miRNAs due to the limited number of proteins, long RNAs and metabolites identified to date [17]. A subset of miRNAs that are expressed in skeletal muscle is increased relative to other tissues and may regulate intrinsic muscle processes is called ‘MyomiR’ [18]. Because unbiased identification of the relative abundance of specific miRNAs in healthy human skeletal muscle has not been published, there is no consensus on the exact definition of MyomiRs [19]. However, current studies have shown that MyomiR is an important regulator of muscle phenotype, and provide the muscle morphology, composition and potential biomarkers of power, especially the miRNA‐1, the miRNA‐133‐a, the miRNA‐133‐b and miRNA‐206, which play key roles in muscle function [15].

In terms of the range of action, soluble myokines mainly regulate multiple organs and systems through the bloodstream but tend to have limited regulatory effects beyond the blood‐tissue barriers [1, 11, 12]. In contrast, MyoEVs tend to act more locally, but have a broader complexity of effects, even capable of crossing blood–brain or blood–testis barriers to transmit complex cellular information and regulate cellular functions at a distance [20]. This characteristic gives them a unique advantage in physiological and pathological processes involving multi‐organ interactions. For example, in neuromuscular diseases or metabolic syndrome diseases, they may play a role by regulating the communication between the central nervous system and peripheral tissues.

### Comparison of Function and Action Mechanism

There are certain functional differences between myokines and MyoEVs. Although only a limited number of myokines have been assigned specific functions, current research has established that myokines can influence a variety of biological processes, including cognition, lipid and glucose metabolism, browning of white adipose tissue, bone formation, endothelial cell function, hypertrophy, skin structure and tumour growth [12]. MyoEVs are primarily involved in cellular communication, not only facilitating intramuscular communication among similar cells [21, 22], but also promoting inter‐organ communication, particularly between muscle and bone [17, 23, 24, 25]. Furthermore, the contents of MyoEVs plays an essential role in mediating interactions within muscle tissue and between muscle and bone [7].

MyoEVs and myokines usually have consistent or synergistic effects on muscle repair, regeneration and metabolic homeostasis, but they may also show completely different effects on other identical organs. For example, for bone tissue, studies have shown that MyoEVs and myokines are both involved in the regulation of bone tissue remodelling and health maintenance. Among them, MyoEVs have been shown to play a positive effect on bone health and remodelling. MiR‐27a‐3P in MyoEVs can activate the β‐catenin pathway and promote the differentiation of pre‐osteoblasts MC3T3‐E1 into osteoblasts [26]. Prrx2 can upregulate MIR22HG and activate the Hippo pathway to promote osteogenic differentiation of BMSCs and alleviating osteoporosis in mice [27]. Studies have also shown that MyoEVs can effectively reverse disuse osteoporosis in mice by enhancing bone formation and inhibiting bone resorption [28]. Some myokines have a negative regulatory effect on bone formation. IL‐6 enhances the generation and differentiation of osteoclasts through RANKL‐dependent mechanisms [29]. Myostatin increases the expression of osteoclast‐related genes, including integrin αVβ3, DC‐STAMP and the calcitonin receptor, reducing bone formation and enhancing bone resorption [30].

### Differences in Clinical Applicability

#### Direct Treatment of Diseases

Myokines and MyoEVs both have the ability to regulate metabolism, cell differentiation and regeneration, thus holding a promising prospect for the treatment of myopathies and other diseases, as well as for improving metabolic homeostasis [11, 21, 31, 32, 33, 34, 35]. Natural myokines and MyoEVs secreted directly by muscles are mixtures of complex components, although a large number of components of myokines and MyoEVs have been identified [11, 36]. The secretome of skeletal muscle cannot be directly used in disease treatment applications due to limitations in analytical and purification technologies [37, 38], and it is almost impossible to simulate the natural secretome through a combination of recombinant proteins [37, 38]. Thus, only single‐component myokines have been used to treat diseases in clinical trials. Single‐component myokines may not be as comprehensively effective as the natural secretome mixtures, but the advantage is that the components are more clearly defined, more stable and also conducive to dose escalation trials for different diseases. This simplicity is beneficial for drug production and also conducive to the conduct and approval of clinical trials.

In contrast to the clearly defined single‐component myokines, MyoEVs derived from skeletal muscles, muscle organoids or myoblasts usually contain a mixture of proteins, microRNAs and mitochondrial DNA [21, 31, 33, 34, 35]. This natural composition may include more novel molecules that are beneficial to health, and the complex composition may simultaneously improve the health of several organs through multiple signalling pathways, possibly with synergistic benefits. However, due to the complexity of MyoEVs' components, their precise analysis and purification are technically challenging. This limits their functional research and clinical applications. Meanwhile, although with the aid of EV extraction techniques, it is easy to crudely obtain a large amount of MyoEVs from the culture medium of skeletal muscle cells [4, 21, 39], some key components may be lost or their activity may be affected due to technical variations, all of which hinder the full utilisation of EVs in applications.

The mass production of MyoEVs presents other technical challenges. Similar to myokines, the composition of naturally secreted MyoEVs is largely contingent upon the condition of the skeletal muscle cells at the time of secretion, which varies with passaging and culture conditions, but significantly influences their potential health benefits. When they are in a state of inflammation, damage, disease, or aging, the harmful components in MyoEVs may increase [40, 41]. Therefore, the health status of the skeletal muscle cells used as secretory devices is extremely important. However, long‐term maintenance of skeletal muscle cells, which serve as the source of MyoEVs, is challenging in vitro. The current difficulty in expanding MuSCs in vitro is that it is impossible to maintain their stemness for a long time, and senescence or differentiation after multiple passages is almost inevitable, leading to a limited lifespan of ~20 passages. Our previous studies based on Lin28a + mouse MuSCs led to a minimal combination of LIN28A, TERT and sh‐p53 (LTS) [42, 43, 44], all of which play important roles during embryonic limb development. The LTS combination can delay the aging of adult muscle progenitor cells, significantly increase their passaging limit in vitro, and greatly improve the usability of MuSCs as MyoEV secretory devices.

#### Drug Delivery Vehicles

Unlike myokines that are directly secreted into the extracellular space, MyoEVs possess a lipid bilayer membrane structure, which makes the various substances they contain less susceptible to degradation, thus they can act as drug delivery vehicles [45, 46, 47, 48, 49]. Moreover, MyoEVs exhibit good biocompatibility, non‐cytotoxicity and low immunogenicity. They are also easy to store, have a long shelf life, high cargo loading capacity and can even cross the blood–brain barrier to enter the central nervous system [50, 51, 52, 53, 54]. Based on these advantages, in recent years, the use of EVs as carriers in the treatment of inflammation, metabolic disorders and in mediating vaccination and drug delivery has seen rapid development [50, 51, 52, 53].

Currently, preclinical testing of EVs as carriers for delivering miRNA or siRNA focuses on tumour diseases such as breast cancer, glioma and pancreatic cancer [10, 55, 56, 57, 58, 59, 60]. In clinical cases, cancer patients often face the risk of cachexia due to their disease. Using MyoEVs as carriers for cancer treatment not only safely delivers antitumor drugs but also improves skeletal muscle and overall metabolism due to their own and contained myokines, providing a more comprehensive treatment strategy, which can be considered a double benefit.

However, there are still unresolved issues in using MyoEVs as drug delivery vehicles. The targeting of MyoEVs in drug delivery needs further exploration, with current methods primarily involving systemic administration and local injection. Additionally, the sustained release of drugs delivered by MyoEVs requires further optimization. At the same time, apart from the difficulty in maintaining the stemness of MuSCs that secrete MyoEVs in vitro, as mentioned earlier, the complex ethical reviews for transgenic cells also limit the development of this technology.

## Conclusion

In summary, MyoEVs and myokines are two crucial bioactive substances secreted by skeletal muscle, exhibiting both similarities and distinctions. MyoEVs are phospholipid bilayer vesicles containing proteins, lipids and RNA, capable of local or distant regulation through mechanisms like endocytosis. Myokines, on the other hand, are proteins or peptides that rely on blood transport to exert systemic or localised effects. Clinically, MyoEVs hold greater potential in disease treatment due to their ability to act as drug carriers and cross barriers such as the blood–brain or blood–testis barriers. Extracted MyoEVs can not only be directly applied to the treatment of specific diseases but also achieve precision medicine by modifying the structure of MyoEVs, altering components and adjusting the ratios of components.

## Author Contributions

X.L., Z.Y., L.Z. and N.S.‐C. designed and wrote the manuscript.

## Conflicts of Interest

The authors declare no conflicts of interest. Ng Shyh‐Chang is an Editorial Board member of Cell Proliferation and a co‐author of this article. He was excluded from the editorial decision‐making related to the acceptance of this article for publication in the journal.

## Data Availability

Data sharing is not applicable to this article as no new data were created or analyzed in this study.
